# Maternal and Neonatal Outcomes of Immune Thrombocytopenia in Pregnancy: A Five‐Year Single‐Center Cross‐Sectional Study

**DOI:** 10.1002/hsr2.71876

**Published:** 2026-06-11

**Authors:** Sara Elkorashi, Hassan Magboul, Abdulrahman F. Al‐Mashdali, Mohammed Alnajjar, Naela AlMallahi, Elmustafa Abdalla, Dina Sameh Soliman, Sara Aldali, Manar Elsheikh, Honar Cherif, Shehab F. Mohamed

**Affiliations:** ^1^ Department of Hematology, National Center for Cancer Care and Research Hamad Medical Corporation Doha Qatar; ^2^ Internal Medicine Department Hamad Medical Corporation Doha Qatar; ^3^ Obstetrics and Genecology Department Hamad Medical Corporation Doha Qatar; ^4^ College of Heath and Science Qatar university Doha Qatar

**Keywords:** Immune thrombocytopenia, ITP, maternal complications, neonatal outcomes, pregnancy

## Abstract

**Objectives:**

Immune thrombocytopenia (ITP) during pregnancy presents unique management challenges. This study represents the first analysis of ITP outcomes during pregnancy in Qatar, aiming to assess maternal and neonatal outcomes at a tertiary care center.

**Methods:**

A 5‐year retrospective study (2018–2022) was conducted at Hamad Medical Corporation, analyzing 81pregnant patients with ITP. Data collection included demographics, hematological parameters, complications, management and treatment responses.

**Results:**

Among the total cohort, 21 patients had chronic ITP, 47 had pre‐pregnancy ITP, and 34 developed ITP during pregnancy. Median platelet counts were 44 × 10⁹/L at diagnosis, with trimester‐specific counts of 36 × 10⁹/L, 44 × 10⁹/L, and 46.5 × 10⁹/L for the first, second, and third trimesters, respectively. Maternal complications were observed in 19.8% of cases, most commonly premature rupture of membranes (4.9%). Neonatal complications affected 12.4% of cases with preterm births (3.8%) and intrauterine growth restriction (3.8%) being the most frequent. The majority (80%) of pregnancies were uncomplicated. Pregnancy‐onset ITP was associated with a higher complication rate (28.5%) compared to pre‐pregnancy ITP (23.2%).

**Conclusions:**

While the majority of pregnancies with ITP are uncomplicated, pregnancy‐onset ITP show a higher complication rates compared to pre‐pregnancy ITP. These findings provide valuable insights into the management of ITP during pregnancy within the Middle Eastern population.

## 1 Introduction

Autoimmune diseases are more prevalent in young women, particularly during their childbearing years. Immune thrombocytopenia (ITP) is an autoimmune disorder associated with increased platelet destruction and impaired platelet production, attributed to antiplatelet autoantibodies. ITP can be primary or secondary to other diseases, including autoimmune diseases, infection, or hematological malignancies. The most common presentation is bleeding, typically mucocutaneous or mucosal. The most serious complication is intracranial hemorrhage, which, although rare, appears to be slightly more common in adults compared to children [1].

ITP can affect pregnant women. Data have shown a 10‐fold increase in the incidence of ITP compared to the general population, with approximately 1 to 3 cases in 10,000 pregnancies [2, 3]. Some data showed a more severe degree of thrombocytopenia in pregnant patients with pre‐existing ITP [4].

Complications can arise from severe thrombocytopenia during pregnancy, such as postpartum hemorrhage and neonatal thrombocytopenia. However, a prospective cohort study conducted by Guillet S et al. showed that pregnant women with ITP do not increase their risk of severe bleeding compared to non‐pregnant women [5]. Moreover, a recent meta‐analysis by Hong Zhang et al. looking at maternal and neonatal outcomes of ITP during pregnancy revealed a risk of bleeding whether antepartum and postpartum hemorrhage occuring in only 0.16% of pregnant patients. Notably, there was no significant difference in bleeding risk between those with pre‐existing ITP and those diagnosed during pregnancy. The 2019 International ITP Consensus Report recommends initial therapy with steroids or intravenous immunoglobulin (IVIG), and in refractory cases, thrombopoietin receptor agonists (TPO‐RAs) or rituximab may be considered, as treatment options, although data on their safety and efficacy in pregnancy remain limited. The meta‐analysis published in 2024 by Hong Zhang et al. estimated that about 0.63% of pregnant patients with ITP required treatment [6].

Managing ITP during pregnancy poses unique challenges. Despite the importance of understanding these challenges, data on ITP and its associated outcomes during pregnancy in the middle east area are rare, and existing reports often present conflicting results [7]. We conducted a retrospective study to evaluate maternal and neonatal outcomes in pregnant patients diagnosed with ITP in Qatar, a country with a young, multiethnic population and access to free maternal healthcare.

## 2 Methods

### 2.1 Study Design and Setting

This was a retrospective, single‐center, cross‐sectional study conducted at Hamad Medical Corporation (HMC), the main tertiary referral center for hematology and obstetrics in Qatar. The study covered a 5‐year period from January 2018 to December 2022.

### 2.2 Case Identification and Eligibility

We identified pregnant women with immune thrombocytopenia (ITP) using a systematic search of the electronic medical record system (Cerner®) at HMC. Inclusion criteria required a confirmed diagnosis of ITP, defined as a platelet count < 100 × 10⁹/L in the absence of other identifiable causes (e.g., preeclampsia, gestational thrombocytopenia, infections, or drug‐induced thrombocytopenia), as per International Working Group (IWG) criteria. All diagnoses were independently reviewed and adjudicated by hematologists. Only singleton pregnancies were included.

We defined chronic ITP as cases where the diagnosis had been established for 12 months or more. Patients diagnosed within less than 1 year before pregnancy were classified as having recently diagnosed ITP, while those diagnosed for the first‐time during pregnancy were categorized as having pregnancy‐onset ITP.

### 2.3 Data Collection

We extracted maternal demographic data, obstetric history, ITP‐related parameters (baseline platelet counts, treatments received), and obstetric/neonatal outcomes. Obstetric complications (e.g., preterm labor, premature rupture of membranes, IUGR) were defined based on institutional obstetric guidelines and obstetrician documentation. Bleeding events were classified using the World Health Organization (WHO) bleeding scale. Data on mode of delivery and use of neuraxial anesthesia were also collected.

Neonatal outcomes included birth weight, and platelet counts within the first 72 h of life. Neonatal thrombocytopenia was defined as a platelet count < 150 × 10⁹/L, with further subclassification for counts < 50 × 10⁹/L. All neonatal complications were recorded.

### 2.4 Statistical Analysis

Descriptive statistics were used to summarize patient characteristics. Categorical variables were expressed as frequencies and percentages (with numerators and denominators); continuous variables were reported as means with standard deviations or medians with interquartile ranges, depending on data distribution.

Comparative analyses between subgroups (e.g., pre‐existing vs. pregnancy‐onset ITP) were performed using Chi‐square or Fisher's exact tests for categorical variables. All statistical tests were two‐sided, and a *p*‐value < 0.05 was considered statistically significant. Analyses were performed using IBM SPSS Statistics, version 28.0.

### 2.5 Ethical Considerations

The study received institutional ethics approval (HMC Protocol MRC‐01‐22‐219). In accordance with IRB approval, the requirement for individual patient consent was waived due to the retrospective nature of the study using de‐identified data.

## 3 Results

### 3.1 Patient Characteristics

The study included 81 pregnant women with a mean age of 29.5 years. The ethnic distribution comprised Middle Eastern (43.3%), Asian (28.3%), and African origin (24.7%), with Qatari nationals representing 22.2% of the total cohort. Primigravida constituted 35.8% of the cohort. Of the total cases, 21 patients had chronic ITP, while 47 had pre‐pregnancy ITP, and 34 developed ITP during pregnancy. Associated autoimmune conditions were present in 30.6% of patients, including antiphospholipid syndrome, systemic lupus erythematosus, Sjogren's syndrome, and thyroid diseases (Table 1). In our cohort, 24 out of 81 patients (29.6%) underwent cesarean section. Neuraxial analgesia (epidural or spinal anesthesia) was administered in 23 patients (28.3), all of whom had platelet counts of ≥ 80 × 10⁹/L at the time of the procedure, in accordance with institutional anesthesiology guidelines.

**Table 1:** Baseline demographic and clinical characteristics of the study population (*n* = 81).

Age, mean (Standard deviation)	29.5 (5.3)
**Nationality, *n* (%)**	
Middle eastern	35 (43.3)
Asian	23 (28.3)
African	20 (24.7)
Others	3 (3.7)
**Primigravida, *n* (%**)	
No	52 (64.2)
Yes	29 (35.8)
**Comorbidity, *n* (%)**	
Antiphospholipid syndrome	3 (3.7)
Autoimmune thyroiditis	1 (1.2)
Asthma	1 (1.2)
Beta thalassemia	2 (2.46)
Progressive familial intrahepatic cholestasis	1 (1.2)
Celiac disease	1 (1.2)
Chronic ITP	21 (25.92)
Common variable immunodeficiency	1 (1.2)
Diabetes mellitus	4 (4.93)
Epilepsy	1 (1.2)
Familial thrombocytopenia	1 (1.2)
Grave's disease	2 (2.46)
Gestational diabetes	6 (7.4)
Hemolytic anemia	1 (1.2)
Hypothyroidism	14 (17.28)
Hypertension	2 (2.46)
Thrombocytopenia during pregnancy	1 (1.2)
Miscarriages	1 (1.2)
Sjogren syndrome	2 (2.46)
SLE	2 (2.46)
None	29 (35.8)
**Multiple pregnancy, *n* (%)**	
No	78 (96.3)
Yes	3 (3.7)

### 3.2 ITP Related Outcomes

**Figure 1:**
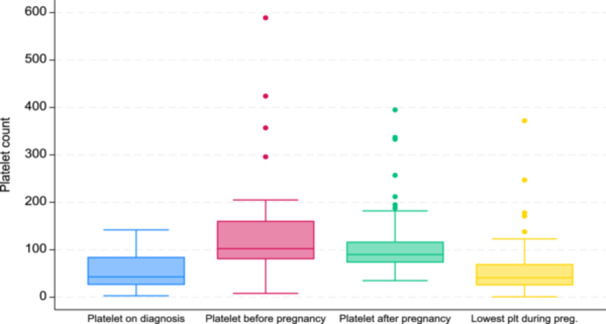
Platelet count trends during pregnancy for patients with ITP, illustrating median platelet counts at diagnosis, in each trimester, and post‐partum.

Analysis of platelet counts throughout pregnancy revealed dynamic changes, with an initial median count of 43 × 10⁹/L at ITP diagnosis, declining to a nadir of 41 × 10⁹/L during pregnancy, compared to pre‐pregnancy levels of 102 × 10⁹/L and post‐pregnancy levels of 90 × 10⁹/L. Trimester‐specific analysis showed median platelet counts of 36 × 10⁹/L, 42.5 × 10⁹/L, and 28 × 10⁹/L for the first, second, and third trimesters, respectively. Notably, patients with chronic ITP maintained higher median platelet counts during pregnancy (44 × 10⁹/L) compared to those with newly diagnosed ITP during pregnancy (36 × 10⁹/L), suggesting potential differences in disease behavior between these two groups (Tables 2, 3) (Figure 1).

**Table 2:** Platelet count outcomes in different time point (count × 10⁹/L).

	Median	IQR	Minimum	Maximum	*n*
Platelet on diagnosis	43	59	3	142	51
Platelet before pregnancy	102	81	8	589	58
Platelet after pregnancy	90	44	35	395	77
Lowest platelet during pregnancy	41	45	1	372	81

**Table 3:** Lowest platelet during pregnancy by gestational age and trimester (count× 10⁹/L).

	Median	IQR	Minimum	Maximum	*n* (%)
**Gestational age trimester** a					
First trimester (≤ 13 weeks)	36	57	10	119	11 (13.75)
Second trimester (14– ≤ 27 weeks)	42.5	34	9	138	26 (32.50)
Third trimester (28 + weeks)	38	54	1	372	43 (53.75)

^a^ 1 missing observation

### 3.3 Maternal and Neonatal Outcomes

Maternal and neonatal outcomes were generally favorable, with 80.2% (65/81) of patients experiencing no maternal complications. Premature rupture of membranes was the most frequent maternal complication, occurring in 4.9% (4/81) of patients, followed by hemorrhagic events (antepartum or postpartum) reported in 3 patients (3.7%) with platelet counts below 34 × 10⁹/L. Miscarriage occurred in 3.8% (3/81) of cases, all in patients with severely low platelet counts of 10 × 10⁹/L.

Regarding neonatal complications, 12.4% (10/81) of neonates experienced at least one adverse event. Intrauterine growth restriction (IUGR) was the most common, occurring in 3.8% (3/81) of cases and associated with maternal platelet counts around 41 × 10⁹/L. A single case of intrauterine fetal demise (1.2%, 1/81) occurred in a patient with a relatively normal platelet count of 138 × 10⁹/Land single case of neonatal thrombocytopenia also 1.2%

Pregnancy‐onset ITP was associated with a higher complication rate (28.5%) compared to pre‐pregnancy ITP (23.2%), although this difference did not reach statistical significance (*p* = 0.08).

### 3.4 Treatment

The majority of ITP patients (54.3%) did not require therapeutic intervention. Among those requiring treatment, combination therapy with steroids and intravenous immunoglobulin (IVIG) was the predominant strategy, administered to 14 patients (17.3%), resulting in significant post‐pregnancy platelet count elevation (up to 337 × 10⁹/L). Notably, thrombopoietin receptor (TPO‐R) agonists were successfully employed in two patients (Both patients were on Eltrombopag before pregnancy and during pregnancy they had refractivness to IVIG and steroids so were started on romiplostim) demonstrating an alternative treatment option in selected cases.

## 4 Discussion

Thrombocytopenia represents the second most common hematological abnormality during pregnancy [8], presenting unique challenges in maternal care. According to The International Working Group (IWG) criteria, ITP is defined by platelet counts below 100 × 10⁹/L and occurs in less than 1% of pregnancies [9]. Our cohort exclusively comprised patients with confirmed ITP diagnoses, allowing for comprehensive analysis of disease patterns and management strategies in this specific population.

### 4.1 Platelet Outcomes and ITP Management

Our analysis revealed consistently low median platelet counts throughout pregnancy, with the nadir occurring during the first trimester. This pattern aligns with previous studies documenting pregnancy‐associated platelet decline due to hemodilution, immune modulation, and disease progression [10]. Notably, chronic ITP patients maintained higher median platelet counts during pregnancy compared to newly diagnosed cases, potentially reflecting better disease control through pre‐pregnancy management, earlier interventions, or possibly indicating a more stable disease state in chronic cases. This observation suggests potential benefits of optimizing ITP control before conception, though further research is needed to confirm this hypothesis.

The combination of steroids and IVIG proved highly effective, achieving substantial post‐pregnancy platelet count improvements (up to 337 × 10⁹/L), supporting current first‐line treatment guidelines [11]. This remarkable response reinforces the value of this therapeutic approach in managing ITP during pregnancy. While TPO‐R agonists (TPO‐RAs) were successfully used in two cases, their Category C classification limits widespread use. However, recent literature supports their safety [12, 13, 14, 15], warranting further investigation as alternative options for cases refractory to or contraindicated for standard therapy. The successful outcomes in our TPO‐RA cases contribute to the growing body of evidence supporting their potential role in pregnancy‐associated ITP management.

### 4.2 Maternal and Neonatal Outcomes

Our finding that 80.2% of patients experienced no significant maternal complications corroborates recent findings published in Blood, which demonstrated excellent maternal prognosis and minimal severe bleeding risk during pregnancy [16]. This high rate of uncomplicated pregnancies is particularly encouraging and suggests that with appropriate monitoring and management; most ITP patients can achieve favorable outcomes. Hemorrhagic complications were primarily observed in cases with severe thrombocytopenia emphasizing the importance of maintaining adequate platelet counts, particularly peripartum. This observation underscores the critical threshold for intervention and highlights the need for vigilant monitoring in severe cases.

While complications were more frequent in pregnancy‐onset ITP compared to pre‐existing cases, Premature rupture of membranes and miscarriage emerged as the predominant maternal complications, consistent with established ITP‐associated risks [8]. This pattern might reflect the challenges of achieving rapid disease control in newly diagnosed cases or could indicate a more aggressive disease course in pregnancy‐onset ITP.

Neonatal complications (12.4%) primarily manifested as IUGR, particularly in cases with lower platelet counts. This aligns with prior studies that report a low incidence of severe neonatal thrombocytopenia in infants born to mothers with ITP [17, 18]. In our cohort, neonatal thrombocytopenia was infrequent and generally mild, with no cases of neonatal hemorrhage. However, the occurrence of intrauterine fetal demise in a patient with normal platelet counts suggests the influence of factors beyond thrombocytopenia in determining fetal outcomes. This observation highlights the complexity of pregnancy outcomes in ITP patients and emphasizes the need for comprehensive maternal‐fetal monitoring beyond platelet counts alone [19, 20].

Pregnancy‐onset ITP was associated with a higher complication rate (28.5%) compared to pre‐pregnancy ITP (23.2%) with a *P* value of 0.08 which did not reach statistical significance but rather a trend towards significance if the power of the study was larger.

## 5 Strengths and Limitations

Our study presents several notable strengths, including being one of the largest cohorts examining ITP in pregnancy in Qatar, a country with a young, multiethnic population and access to free maternal healthcare, with comprehensive representation of Middle Eastern demographics and detailed documentation of platelet counts throughout pregnancy and postpartum period. The inclusion of distinct ITP groups pre‐pregnancy, and pregnancy‐onset) with their respective outcomes provides valuable comparative data. However, the study has some important limitations to consider: its single‐center retrospective design may introduce selection bias, the sample size of 81 patients limits statistical power for subgroup analyses, and some neonatal platelet counts were unavailable (in a few cases where newborns were assessed at outside facilities or not immediately tested), which might slightly underestimate the incidence of neonatal thrombocytopenia. Additionally, the lack of local standardized treatment protocols during the study period and potential unmeasured confounding factors such as concurrent medications or comorbidities may impact the interpretation of outcomes. Another limitation of this study is the inclusion of patients with secondary ITP, which may have introduced heterogeneity into the cohort. In particular, comorbid conditions such as systemic lupus erythematosus (SLE) could independently contribute to adverse maternal or neonatal outcomes, making it difficult to isolate the effect of ITP alone. Additionally, the absence of a control group of pregnant individuals without ITP limits our ability to establish a direct causal relationship between ITP and the complications observed. These factors should be considered when interpreting the results. Despite these limitations, our findings may contribute significantly to the understanding of ITP management during pregnancy in the Middle Eastern population and provide a foundation for future prospective, multicenter studies.

## 6 Implications and Recommendations

Early identification and monitoring of ITP in pregnancy are essential, particularly for pregnancy‐onset cases with higher complication rates. Steroids and IVIG remain effective first‐line treatments, while severe thrombocytopenia may require closer monitoring and more aggressive management. The successful use of TPO‐RAs in refractory cases suggests potential alternative therapies. Given the high prevalence of associated autoimmune conditions, a multidisciplinary approach is crucial. Future studies should validate these findings and refine existing treatment protocols.

## 7 Conclusion

This study demonstrates that while most pregnant women with ITP can achieve favorable outcomes with appropriate management strategies, patients diagnosed during pregnancy face higher complication rates. These findings underscore the critical importance of early recognition, pre‐pregnancy counseling when possible, and the implementation of risk‐stratified management protocols. The results emphasize that successful outcomes depend on vigilant monitoring and prompt intervention, particularly in newly diagnosed cases, highlighting the need for coordinated care between hematology and obstetric teams to optimize maternal and fetal outcomes in this vulnerable population.

## Author Contributions

**Sara Elkorashi:** conceptualization, data curation. **Hassan Magboul:** visualization, writing – original draft. **Abdulrahman F. Al‐Mashdali:** validation, writing – original draft, writing – review and editing. **Mohammed Alnajjar:** methodology. **Naela AlMallahi:** conceptualization, data curation. **Elmustafa Abdalla:** writing – original draft. **Dina Sameh Soliman:** methodology. **Sara Aldali:** writing – original draft. **Manar Elsheikh:** formal analysis, validation, visualization. **Honar Cherif:** supervision, validation. **Shehab F. Mohamed:** conceptualization, data curation, supervision, validation.

## Ethics Statement

This research project received ethical approval from the Medical Research Center at Hamad Medical Corporation (protocol MRC‐01‐22‐219). Since the analysis utilized only publicly accessible data, individual patient consent was not necessary. “Dr Sara Elkorashi affirms that this manuscript is an honest, accurate, and transparent account of the study being reported; that no important aspects of the study have been omitted; and that any discrepancies from the study as planned have been explained.”

## Consent

All authors have read and approved the final version of the manuscript. Abdulrahman F. Al‐Mashdali had full access to all of the data in this study and takes complete responsibility for the integrity of the data and the accuracy of the data analysis.

## Conflicts of Interest

On behalf of all authors, the corresponding author states that there is no conflict of Interest.

## Transparency Statement

The lead author Abdulrahman F. Al‐Mashdali affirms that this manuscript is an honest, accurate, and transparent account of the study being reported; that no important aspects of the study have been omitted; and that any discrepancies from the study as planned (and, if relevant, registered) have been explained.

## Data Availability

All data generated or analyzed during this study are included in this article. Further enquiries can be directed to the corresponding author.
